# Long-Term Effect of Cognitive Behavioral Therapy in Managing Subclinical Depression: A Systematic Review and Meta-Analysis

**DOI:** 10.1155/da/1610909

**Published:** 2025-08-15

**Authors:** Raffy C. F. Chan, Ming Chen, Jacqueline L. M. Chan, David H. K. Shum, Yuan Cao

**Affiliations:** ^1^Department of Rehabilitation Sciences, Hong Kong Polytechnic University, Hung Hom, Hong Kong SAR, China; ^2^Department of Social Work and Social Administration, University of Hong Kong, Pokfulam, Hong Kong SAR, China

## Abstract

Recent research has emphasized the continuum of depression, highlighting the significance of early intervention for subclinical depression. However, previous studies often focused on specific populations or lacked comparisons across participants and intervention characteristics in the effectiveness of cognitive behavioral therapy (CBT). This systematic review and meta-analysis (CRD42024498284) aimed to address these gaps by examining the effectiveness of CBT in managing subclinical depression and its potential for preventing the transition to major depression. A comprehensive search across seven databases from inception to March 2025, identified 23 randomized controlled trials (RCTs) involving 5877 participants. Meta-regression, sensitivity analysis, and funnel plots were utilized to assess heterogeneity, publication bias, and study quality. CBT significantly improved subclinical depressive symptoms (at postassessment: *g* = −0.89; 95% confidence interval (CI) = −1.57 to −0.20 and follow-up: *g* = −0.56; 95% CI: −0.93 to −0.18) and anxiety symptoms (at postassessment: *g* = −0.92; 95% CI: −1.84 to −0.00 and follow-up: *g* = −0.70; 95% CI: −1.15 to −0.25), but had no notable impact on quality of life. Meta-regression analysis identified the number of CBT sessions as factors influencing CBT effectiveness in managing depressive symptoms. While there are statistically significant results (RR = 0.62; 95% CI = 0.50–0.77) indicating CBT's preventive efficacy in transitioning from subclinical to major depression, evidences were limited by the self-reporting data. The majority of included studies came from Europe which limited generalizability, and comparisons between different types of CBT, education levels, and CBT components were limited. In general, CBT has been demonstrated to be effective in managing depressive symptoms over time. Additional research, particularly from diverse regions and comparative studies between CBT and alternative treatments, is imperative to overcome the current study's limitations.

## 1. Introduction

Depression is one of the most common mental disorders and its economic burden on public health services globally is substantial. In research and clinical settings, symptoms of depression are used to diagnose depressive disorders based on two diagnostic systems, namely, the Diagnostic and Statistical Manual of Mental Disorders Fifth Edition (DSM-5) and the International Classification of Disease 11^th^ Edition (ICD-11). These diagnostic systems require the presence of the number, duration, and severity of depressive symptoms to meet a certain standard before a formal diagnosis can be made to deliver standardized and appropriate treatment. However, due to the strict diagnostic criteria, people with depressive symptoms, but without meeting the criteria might be overlooked. Recent studies suggested that depression should be better understood as a continuum due to the complexity and clinically relevant condition of depressive symptoms [1], thus, increasing attention to better understand the subclinical depression to develop appropriate treatment as a prevention strategy for the development of depression.

Subclinical depression (also known as early-onset, subsyndromal, or subthreshold depression), proposed by Judd [2], is characterized by the presence of any two or more depressive symptoms (depressed mood or the loss of interest or pleasure must be present) for at least 2 weeks with significant impairment without meeting the criteria of other depressive disorders. However, it is important to note that the definition of subclinical depression still varies among researchers [3]. Despite the heterogeneous definitions of subclinical depression, it is widely defined by researchers as a clinically relevant depressive symptom based on: (1) depression measurement tools used in clinical interviews and (2) not meeting the diagnostic criteria for major depression following the DSM-5, or the presence of any depressive episode (ICD-10) [3, 4]. Although subclinical depression is not widely accepted by all researchers, in recent years, research on the subclinical depression population has been gradually increasing due to its potential capability to understand the development of major depression [5–7].

Subclinical depression is highly prevalent, but the reported prevalence varies depending on the different diagnostic criteria used. A recent review [8] highlighted the significant difference in the prevalence of subclinical depression based on different diagnostic criteria: 9.97% when using diagnostic manuals (such as DSM or ICD systems) and 13.41% when relying on other measurements such as the use of self-reported questionnaires. It also suggested that based on 113 studies with subclinical depression populations across 39 countries, the prevalence is estimated to be approximately 11%. Females are more likely to experience subclinical depression than males, and age also plays a significant role with youth (under 18) reported to have the highest prevalence at 14.17%, followed by older adults (above 60) at 12.95% and adults (between 18 and 60 years old) at 8.92%. Because of the mild depressive symptoms, individuals with subclinical depression might be overlooked and the overall prevalence of subclinical depression might be underrepresented. With its high prevalence, individuals with subclinical depression are reported to have significantly greater use of health care services compared to the nondepressed [9], and the cost of managing subclinical depression could be comparable with those with major depression [10]. It is reported that individuals with subclinical depression are approximately three times more likely to transition to major depression compared to nondepressed individuals [8]. In addition, previous studies stated that younger age, low education, and substance use significantly increase the risk of converting from subclinical depression into major depression [11]. Furthermore, subclinical depression is associated with a high risk of developing into long-term disorders, including the conversion to other depressive disorders, co-occurring generalized anxiety disorders (GADs), substance use, and an increased risk of suicide [12]. Previous studies have indicated that even with mild depressive symptoms, individuals with subclinical depression will also experience an impact on their quality of life, especially when depressive symptoms are comorbid with other health-related diseases, such as diabetes [13, 14], and persistent back pain [15]. In addition, previous worldwide studies indicated the high prevalence of co-occurring depression and anxiety, with 45.7% of individuals with lifetime major depression disorder experiencing a lifetime history of one or more anxiety disorders [16]. Most importantly, the presence of significant anxiety symptoms will lead to an increase in the severity of depression, more role impairment, and more suicidal thoughts in individuals with major depressive disorder [17, 18]. These, in turn, increase the burden on an individual's well-being and quality of life. The World Health Organization (WHO) defines prevention as any approaches and activities that aim to reduce the impact of a disease or disorder on the individual, stopping or slowing down the progression, or reducing the disability brought by the disease or disorder [19]. Therefore, understanding and identifying effective treatments for managing subclinical depression is of paramount importance.

Although pharmacological therapy such as the use of antidepressants is recommended to treat depression, results from previous meta-analyses found that the use of antidepressants and benzodiazepines might not be appropriate in managing subclinical depression [20]. With the presence of mild depressive symptoms, pharmacological therapy might overtreat patients with subclinical depression. The use of antidepressants in the usual treatment among patients with minor depression showed small clinical outcomes [21]. In addition, because depression is characterized by remission and relapses, using antidepressants for a long period might also come with its drawbacks and side effects. Hence, it is important to examine the effectiveness of nonpharmacological treatments as effective management for subclinical depression.

The National Institute for Health and Care Excellence (NICE) guidelines (2022) recommend a range of psychological interventions and treatments for managing less severe depression, including cognitive behavioral therapy (CBT), group behavioral activation, group mindfulness and meditation, group exercise, interpersonal psychotherapy, counseling, and short-term psychodynamic psychotherapy. Among these recommendations, guided self-help interventions, particularly those based on CBT and behavioral activation, have demonstrated effectiveness and are recommended as first-line treatment for individuals with less severe depression [22]. Previous studies have examined the efficacy of nonpharmacological treatment in managing subclinical depression, and it is suggested that psychotherapy, especially CBT, might be the most effective intervention for subclinical depression [23]. CBT is one of the most widely used and effective evidence-based psychotherapies for managing depression through targeting maladaptive thoughts and negative appraisal of life events which plays an important role in the development of depression [24]. CBT comprises multiple components, including homework, psychoeducation, and problem-solving that can be performed in different combinations and platforms [25]. Originally designed to be a face-to-face individual-based therapy, group-based CBT (g-CBT) was developed for mass delivery, and its effects were found to have no significant difference compared to individual-based CBT in managing depression [26]. The g-CBT was tested to be effective in managing depressive symptoms among older adults with subclinical depression [27]. Due to the high cost, a lack of trained professionals, and the lack of motivation to seek intensive face-to-face treatment in people with depressive symptoms, remote versions of CBT such as internet-based CBT (i-CBT) and telephone-based CBT (t-CBT) that require minimal to no support (self-guide program) have been developed [28, 29] and proved to have a small but significant effect in reducing depressive symptoms in older adults with subclinical depression [30].

Previous network meta-analysis suggested psychotherapy, especially CBT might be the most effective individualized psychotherapy for managing subclinical depression [23, 31]. Additionally, some review studies found a small to moderate significant effect on subclinical depression using psychotherapy [28], but these studies had several limitations. Some studies were too wide that they covered all types of psychotherapy without focusing specifically on the form or intensity of CBT delivery for managing subclinical depression [1, 28, 29]. On the other hand, some studies were too narrow which only focused on a particular type of participants [23, 30], focused on the short-term effect of CBT [10, 23, 31], or focused solely on the long-term effect of i-CBT on subclinical depression [29].

As one of the most widely used psychotherapies, the effectiveness of CBT should be further examined to determine its feasibility and implications for managing subclinical depression in a long period. This systematic review and meta-analysis aims to address prior limitations and inform future clinical practice in managing subclinical depression. It evaluates the effectiveness of CBT compared to other therapeutic approaches and examines differences between i-CBT and traditional face-to-face CBT. There were two core objectives:

1. To primarily assess the long-term effectiveness of CBT in reducing depressive symptoms in people with subclinical depression compared to the control group. Examine the effectiveness of CBT in preventing the development of major depression and provide the comprehensive examination including participants' and study characteristics on the use of CBT in managing subclinical depression. Furthermore, this study also compare the effectiveness among various types of CBT approaches, especially between i-CBT and other forms of CBT approaches in managing subclinical depression.
2. To examine the effects of CBT on GAD (anxiety symptoms) and quality of life as a secondary outcome.

## 2. Method

This systematic review and meta-analysis follow the Preferred Reporting Items for Systematic Review and Meta-analysis (PRISMA) guidelines and has been registered at the PROSPERO International Prospective Register of Systematic Reviews (CRD42024498284). All the included studies were approved by their ethics committees or institutional review boards.

### 2.1. Search Strategy

The literature search was conducted on seven databases, including PubMed, Scopus, Embase, Medline, PsycINFO, CINAHL, and Web of Science from inception to March 17, 2025, following the participants, intervention, comparison, outcome, and study design (PICOS) framework using specific terms on two concepts: subclinical depression and CBT, such as “subthreshold depression,” “early-onset depression,” “cognitive–behavioral treatment,” and “CBT.” More details on the search keywords and terms are presented in the Supporting Information 1: Used in Search of Studies.

### 2.2. Selection Criteria

Studies were included if: (1) they are published in English, (2) participants must be explicitly included as having subclinical depression either through a diagnostic interview with a well-developed depression scale and without the presence of major depression disorder categorized by the DSM-5 or ICD-10 (proposed by Judd [2]), (3) randomized controlled trials (RCTs) conducted with CBT, (4) conduct a follow-up assessment of the participant for at least 1 month and report the results of the follow-up assessment, and (5) contain reported changes in symptoms assessed by standardized and well-developed depression, anxiety, and quality of life scale (Supporting Information 1: Inclusion and Exclusion Criteria). The current systematic review and meta-analysis also included studies with participants who were diagnosed with major depressive disorder, but only when results were specifically reported for participants with subclinical depression. Studies from the same author that shared identical study design and participants' demographic characteristics will be considered as nonindependent studies in the current systematic review and meta-analysis.

### 2.3. Data Extraction

All included studies were screened and extracted by two independent researchers (Raffy C. F. Chan and Ming Chen) beginning with titles and abstracts, followed by full-text screening following the inclusion criteria listed above, any discrepancies would be resolved through discussion or consultation with Yuan Cao. The procedure of the screening process was recorded, including the reasons for exclusion.

All used data was extracted by two independent researchers (Raffy C. F. Chan and Ming Chen), including the years and country of the studies, number and demographic characteristics of the participants (age and gender of both intervention group and control group; associated physical disease for participants), types of depressive assessment, types of intervention (delivery method and component), depressive symptoms based on established depression scale during baseline, short-term, and long-term (short term: postassessment or assessment conducted less than 1 month after the intervention; long-term: follow-up-assessment conducted more than 1 months after intervention).

Dichotomous variables are represented by risk ratios (RRs) for major depression (intervention and control groups); continuous variables are represented by means and standard deviations of depressive symptoms, anxiety symptoms, and quality of life. In the results of the continuous variable outcomes for all current included studies, a positive change in value implies elevated symptoms of depression or anxiety at the follow-up assessment, whereas a negative change indicates a reduction in symptoms from the initial assessment. For quality of life, it is the opposite. A RR of less than 1 signifies a reduction in major depression incidence due to the CBT intervention in the intervention group, while a ratio greater than 1 indicates an increase.

### 2.4. Quality Assessment

Following the recommendation from the Cochrane Handbook for Systematic Reviews of Interventions, the risk of bias (ROB) was assessed using the ROB tool-2 (ROB 2) [32]. Bias resulted from the randomization process, deviations from intended interventions, missing outcome data, measurement of the outcome, and the selection of the reported results were assessed independently by two independent researchers (Raffy C. F. Chan and Jacqueline L. M. Chan), any discrepancies would be resolved through discussion.

### 2.5. Statistical Analysis

To evaluate the clinical effectiveness of CBT, this meta-analysis recorded major depressive symptoms in both the experimental and control groups at the final follow-up. RRs were computed as the ratio of individuals in each group developing major depressive symptoms to the total group size.

The current meta-analysis will extract the number of participants who transitioned from subclinical depression to major depression reported in each study to calculate RR and number needed to treat (NNT). The current study used the meta package in R (Rx64 4.40) for effect size calculations and constructing forest plots. In this study, RR is viewed as a measure of the strength of association between exposure and outcome. An RR of 1.0 indicates no association; values below 1.0 suggest a potential protective effect, and values above 1.0 suggest increased risk [33]. Furthermore, the NNT translates RR into the number of patients required to achieve a favorable outcome, facilitating the clinical interpretation of the findings. RR and NNT were calculated using Rx64 4.40 software, providing valuable insights into the clinical significance of CBT.

Using the meta package in Rx64 4.40 software, overall effects were determined using random effects models weighted by inverse variance. When a study included multiple forms of CBT intervention groups, data (mean, SD, and *n*) from these groups at the final follow-up stage were combined before calculating the overall effect size based on the Cochrane recommendations [34, 35]. Standardized mean difference (SMD-Hedges's *g* method) assessed between-group differences, with all outcomes presented with 95% confidence intervals (CIs). The interpretation of effect size (*g*) is typically as follows: *g* = 0.2 represents a small effect, *g* = 0.5 is a medium effect, and *g* = 0.8 is a large effect [36]. In the current meta-analysis, a negative combined effect size (*g* < 0) indicates an improvement in depressive symptoms and anxiety symptoms, *g* = −0.5 was considered as a moderate effect size. In contrast, a positive effect size indicates an improvement in quality of life. Statistical heterogeneity was evaluated using the *χ*^2^ test and *I*^2^ statistic, with *I*^2^ values above 50% indicating moderate heterogeneity and exceeding 75% indicating high heterogeneity [37]. The long-term effect of CBT on subclinical depression was assessed by analyzing depressive symptom outcomes at the last follow-up in all included studies for both intervention and control groups.

Studies were considered as outliers if their 95% CI did not overlap with the pooled effect size's 95% CI [38]. However, these outliers were retained if their inclusion did not change the direction of the overall effect or alter the statistical significance of the pooled estimate [39].

Subgroup analysis explored treatment effects and heterogeneity variations among different subgroups. Two subgroup analysis approaches were considered factors influencing CBT's long-term effects. The first focused on participant characteristics (age and gender of both intervention group and control group; associated disease for participants, etc.). The second examined study's characteristics impact CBT efficacy. Additionally, the meta-analysis investigated CBT's long-term effects on participants' quality of life and anxiety. Subgroup analysis would not be performed on certain participants and study characteristics if such characteristics only occurred among less than three studies. For subgroup analyses based on types of CBT approaches, data from distinct CBT intervention groups within the same study were analyzed separately in their respective subgroups. In all other cases, combined data were used to avoid overrepresentation of studies with multiple CBT arms. This study also included all studies comparing CBT with other nonpharmacological psychotherapies, as well as studies comparing i-CBT with other forms of CBT, meta-analyses were conducted for both categories. Depressive symptoms were examined for the presence of publication bias in all studies by the Egger test [40], the trim-and-fill method and the funnel plot [41]. The asymmetric shape of the funnel plot, numbers from the trim-and-fill analysis, and the result of *p* < 0.05 from the Egger test will indicate the presence of publication bias.

### 2.6. Meta-Regression

To address heterogeneity and assess the impact of CBT on controlling subclinical depression, meta-regression analyses were performed using Rx64 4.40 software. The meta-regression models included available continuous variables related to CBT characteristics (duration of intervention and number of CBT sessions) and participant characteristics (mean age). All variables in the meta-regression models underwent analysis using both multivariate and univariate regression models. Variables selected from individual studies should be present and clearly defined in at least 10 studies. Studies lacking relevant variables were excluded from the meta-regression analysis. The estimate represented the regression coefficient of each linear regression model, indicating the slope. The 95% CI was computed for the beta coefficient values. A *p*-value < 0.05 was deemed statistically significant, indicating the predictor's association with the results.

### 2.7. Sensitivity Analysis

Sensitivity analyses were employed to assess individual study effects on depressive symptom outcome heterogeneity, determining if any study significantly influenced the composite outcome (*p* > 0.05). Studies with a notable impact on composite outcome heterogeneity were excluded to evaluate their impact on outcome stability. If changes occurred in the composite outcome (e.g., the significant difference becoming nonsignificant), the study was removed from the final meta-analyses. If no significant change occurred, the study was retained to ensure comprehensive analysis. Sensitivity analyses involved analyzing the combined results' heterogeneity post-exclusion of each study using the leave-one-out method in R x64 4.40. In this study, sensitivity analyses were also conducted based on the ROB 2.0 results assessed independently by two researchers (Raffy C. F. Chan and Ming Chen) test results. The stability of the findings was assessed by comparing changes in heterogeneity and effect size before and after removing high-risk studies.

## 3. Result

### 3.1. Study Characteristics

**Figure 1:**
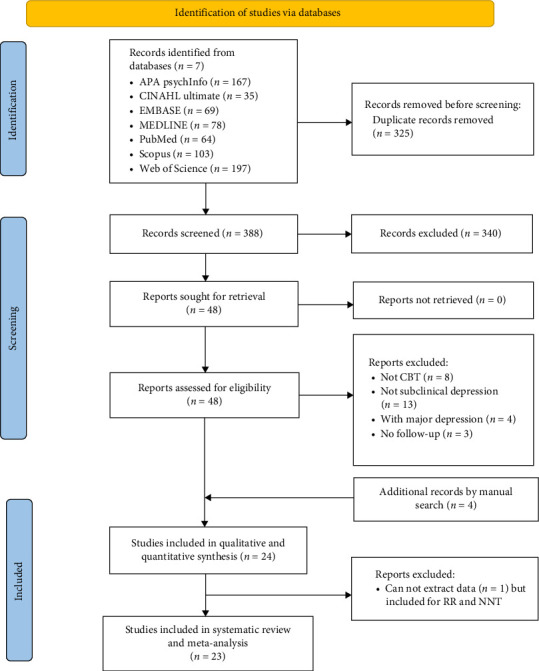
PRISMA flowchart.

The initial search identified 713 articles. After removing 325 duplicates and 388 titles and abstracts screened. Of the 20 full texts screened, an additional four articles from other similar reviews that met our criteria were included. One study was only included for RR and NNT [42]. To avoid bias, nonindependent studies [43, 44] were combined during the current meta-analysis. Thus, the final review consisted of 22 studies from 2001 to 2025, consisting of 5877 participants (sample size ranging from 41 to 846). A detailed description of the selection process is presented in Figure 1.

Within the 23 studies [13–15, 43–62], three major types of CBT approaches were identified, including g-CBT (*n* = 10), i-CBT (*n* = 13), t-CBT (*n* = 2), and individual CBT (*n* = 21). The CBT duration in the included studies could be categorized into three groups, including lasting less than 1 month (*n* = 3), 1–3 months (*n* = 14), and more than 3 months (*n* = 5). The CBT follow-up durations in the included studies could be categorized into three groups, including less than 6 months (*n* = 4), 6–12 months (*n* = 6), and more than 12 months (*n* = 12).

The severity of depression was measured with the Beck Depression Inventory (BDI-II; *n* = 5), Center for Epidemiological Studies Depression Scale (CES-D; *n* = 15), and Patient Health Questionnaire-9 (PHQ-9; *n* = 3). The outcomes were compared with various types of control groups, including treatment-as-usual (*n* = 9), enhanced usual care (*n* = 2), sham treatment (*n* = 1), waitlist (*n* = 6), health education (*n* = 4), physical exercise (*n* = 1), relaxation training (*n* = 1), psychoeducation (*n* = 1), and internet-based psychoeducation (*n* = 1).

The included studies were conducted in Europe (*n* = 12), North America (*n* = 3), Asia (*n* = 7), and Australia (*n* = 1). The characteristics of the included studies, including the participants' demographic characteristics, types of CBT approaches, intervention duration, frequency, and assessment time points are presented in Table 1, and the CBT components of the included studies are presented in Supporting Information 1: Table S1.

### 3.2. Primary Outcome

#### 3.2.1. The Short-Term Effect of CBT on Depressive Symptoms

Fourteen studies examined the short-term efficacy of CBT in managing subclinical depression revealed a large significant effect size (*g* = −0.89; 95% CI = −1.57 to −0.20) with high heterogeneity (*τ*^2^ = 2.04; *p* < 0.01; *I*^2^ = 98%) compared to a control group (treatment as usual or waitlist control).

#### 3.2.2. The Long-Term Effect of CBT on Depressive Symptoms

**Figure 2:**
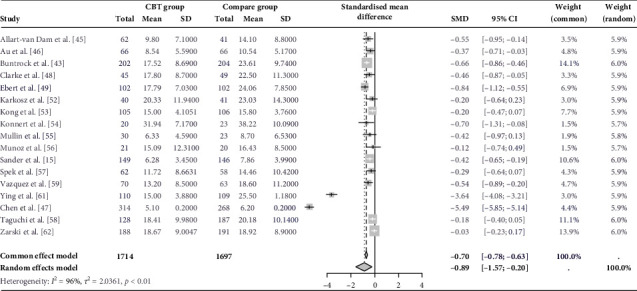
Changes in depressive symptoms during postintervention assessment. CI, confidence interval; SMD, standard mean difference.

Twenty studies examined the long-term efficacy of CBT in managing subclinical depression revealed a moderate significant effect size (*g* = −0.56; 95% CI = −0.93 to −0.18) with high heterogeneity (*τ*^2^ = 0.78; *p* < 0.01; *I*^2^ = 96%) when compared to a control group (treatment as usual or waitlist control; Figure 2).

##### 3.2.2.1. The Subgroup Analysis on Depression Symptoms

Subgroup analyses were performed considering a wide range of participants and study characteristics. Given significant heterogeneity across groups, a random effects model was consistently applied to ensure analytical consistency. Detailed data for all subgroup analyses (participants and study characteristics) are available in Table 2.

##### 3.2.2.2. Participants' Characteristics

*3.2.2.2.1. Mean Age*. In the subgroup analysis based on mean age, significant composite effect sizes were observed only for adults (*g* = −0.63; 95% CI: −1.08 to −0.17) with high heterogeneity (*τ*^2^ = 0.94; *p* < 0.01; *I*^2^ = 97%). Marginal insignificant effects were exhibited in studies for older adults (*g* = −0.27; 95% CI: −0.53 to −0.00) with low heterogeneity (*τ*^*2*^ = 0.02; *p*=0.27; *I*^2^ = 24%).

*3.2.2.2.2*. *Gender*. In the subgroup analyses by gender, the composite effect sizes of studies with more male participants were insignificant (*g* = −0.34; 95% CI: −0.71 to 0.03) with high heterogeneity (*τ*^2^ = 0.08; *p*=0.01; *I*^2^ = 78%). Studies with more female participants exhibited significant effect sizes (*g* = −0.59; 95% CI: −1.05 to −0.13) with high heterogeneity (*τ*^2^ = 0.95; *p* < 0.01; *I*^2^ = 97%).

*3.2.2.2.3. Associated Disease*. Subgroup analyses based on studies targetted on the disease population and studies targetted on the healthy population revealed that both groups exhibited significant effect sizes (*g* = −0.22; 95% CI: −0.36 to −0.08) and (*g* = −0.71; 95% CI: −1.25 to −0.17) with low (*τ*^2^ = 0.01; *p*=0.23; *I*^2^ = 26%) and high (*τ*^2^ = 1.11; *p* < 0.01; *I*^2^ = 97%) heterogeneity, respectively.

##### 3.2.2.3. Study Characteristics

*3.2.2.3.1. Year of Publication*. Subgroup analyses based on the year of article publication revealed that publications from both 10 years ago and the recent 10 years exhibited significant effect sizes (*g* = −0.21; 95% CI: −0.39 to −0.03) and (*g* = −0.74; 95% CI: −1.31 to −0.18) with low (*τ*^2^ = 0.02; *p*=0.15; *I*^2^ = 35%) and high heterogeneity (*τ*^2^ = 1.15; *p* < 0.01; *I*^2^ = 98%) respectively.

*3.2.2.3.2*. *Country of Publication*. Subgroup analyses based on published countries revealed that studies from North America exhibited insignificant effect sizes (*g* = −0.16; 95% CI: −0.45 to 0.14) with low heterogeneity (*τ*^2^ = 0; *p*=0.62; *I*^2^ = 0%), whereas those from Asia showed significant moderate to large effect sizes (*g* = −1.13; 95% CI: −2.22 to −0.05) despite high heterogeneity among the studies (*τ*^2^ = 2.11; *p* < 0.01*; I*^2^ = 99%), and studies from Europe showed significant but small effect size (*g* = −0.27; 95% CI: −0.40 to −0.14) with substantial heterogeneity (*τ*^2^ = 0.03; *p*=0.02; *I*^2^ = 53%).

There was only one study conducted in Australia, thus, it was not included in the current subgroup analysis.

*3.2.2.3.3. Types of CBT Approaches*. In subgroup analyses of the two different types of CBT, only i-CBT demonstrated significant effect sizes ranging from small to large (*g* = −0.81; 95% CI: −1.38 to −0.25) despite high heterogeneity (*τ*^2^ = 0.97; *p* < 0.01; *I*^2^ = 98%). Conversely, g-CBT exhibited insignificant effect sizes (*g* = −0.11; 95% CI: −0.24 to 0.02) with low heterogeneity (*τ*^2^ = 0.01; *p*=0.36*; I*^2^ = 9%).

There were only two studies conducted with t-CBT and two studies with in-CBT, thus, they were not included in the current subgroup analysis.

*3.2.2.3.4. Intervention Duration*. Subgroup analyses based on intervention duration revealed that the group of studies conducted within one to 3 months demonstrated a significant moderate effect size (*g* = −0.69; 95% CI: −1.27 to −0.11) despite high heterogeneity (*τ*^2^ = 1.20; *p* < 0.01; *I*^2^ = 98%).

In addition, studies that conducted less than 1 month and over 3 months exhibited significant effect size (*g* = −0.24; 95% CI: −0.42 to −0.06; *g* = −0.31; 95% CI: −0.45 to −0.16) with low heterogeneity (*τ*^2^ = 0.55; *p*=0.55; *I*^2^ = 0%; *τ*^2^ = 0.27; *p*=0.27; *I*^2^ = 23%), respectively.

*3.2.2.3.5*. *Follow-Up Duration*. Subgroup analyses based on different follow-up durations revealed that the only follow-up group conduct less than 6 exhibited a large significant effect size (*g* = −0.69; 95%CI: −0.90 to −0.48) with low heterogeneity (*τ*^2^ = 0.01; *p*=0.30; *I*^2^ = 18%), while follow-up between 6–12 months and follow-up beyond 12 months exhibited a an insignificant effect (*g* = −0.82; 95% CI: −2.03 to 0.37) and marginal significant but small effect size (*g* = −0.39; 95% CI: −0.78 to 0.00) with high (*τ*^2^ = 2.21; *p* < 0.01; *I*^2^ = 98%; *τ*^2^ = 0.45; *p* < 0.01; *I*^2^ = 96%) heterogeneity, respectively.

*3.2.2.3.6. Attrition Rate*. Subgroup analyses based on different attrition rates revealed that the low to moderate attrition rate group exhibited significant effect sizes (*g* = −0.24; 95% CI: −0.36 to −0.12) with low heterogeneity (*τ*^2^ = 0.02; *p*=0.03; *I*^2^ = 48%). Studies with high attrition rates exhibited significant effect (*g* = −0.98; 95% CI: −1.82 to 0.12) with high heterogeneity (*τ*^2^ = 1.67; *p* < 0.01; *I*^2^ = 98%).

#### 3.2.3. Comparative Analysis

##### 3.2.3.1. CBT vs. Other Forms of Treatment

Four studies examined the effectiveness of CBT compared to other psychological therapies. The results exhibited an insignificant effect (*g* = −0.71; 95% CI = −1.89 to 0.47) with high heterogeneity (*τ*^2^ = 1.43; *p* < 0.01; *I*^2^ = 99%) in managing depressive symptoms among the subclinical depression population (Table 3).

##### 3.2.3.2. i-CBT vs. Other Forms of CBT Approaches

Four studies examined the effectiveness of i-CBT compared to other forms of CBT. The results suggested that there is a small but significant effect favoring i-CBT (*g* = −0.27; 95% CI = −0.41 to −0.13) with low heterogeneity (*τ*^2^ = 0.00; *p*=0.37; *I*^2^ = 4%) in managing depressive symptoms among the subclinical depression population (Table 4).

#### 3.2.4. The RR and NNT for the CBT Prevention

The results of the RR and NNT calculations are presented in Table 5. The overall RR was 0.62 (95% CI = 0.50–0.77; *p* < 0.001), providing evidence that CBT is associated with a statistically significant reduction in the risk of progression to major depression. However, the interpretation of RR in this review should be contextualized, taking heterogeneity across studies and self-reported data included in analysis into account. The corresponding NNT was 8.79 (95% CI = 6.67–14.55), indicating that approximately nine individuals would need to receive CBT to prevent one case of major depression.

### 3.3. Secondary Outcome

#### 3.3.1. The Effects of CBT on Anxiety Symptoms

Ten studies (from 11 articles) examined the efficacy of CBT for changes in anxiety symptoms. The changes in severity of anxiety symptoms was measured with the Beck Anxiety Inventory (BAI; *n* = 1), GAD-7 (*n* = 6), Hospital Anxiety and Depression Scale (HADS-A; *n* = 3), and the State–Trait Anxiety Inventory (STAI; *n* = 1).

The results showed a significant effect size in managing anxiety symptoms post-assessment and at follow-up (*g* = −0.92; 95% CI = −1.84 to −0.003 and *g* = −0.70; 95% CI = −1.15 to −0.25) with high heterogeneity (*τ*^2^ = 2.18; *p* < 0.01; *I*^2^ = 99% and *τ*^2^ = 0.50; *p* < 0.01; *I*^2^ = 97%), respectively (Table 6).

#### 3.3.2. Quality of Life

Three studies examined the efficacy of CBT in quality of life. The changes in the quality of life were measured with the Assessment of Quality of Life (AQoL-6D; *n* = 1), EuroQoL (EQ-5D; *n* = 1), Health Survey 12-Item Short Form-version 2 (SF-12v2; *n* = 1).

The results showed no significant effect size in quality of life (*g* = −0.06; 95% CI = −0.28 to 0.16) with low heterogeneity (*τ*^2^ = 0.02; *p*=0.17; *I*^2^ = 43.7%; Table 7).

### 3.4. Quality Assessment

Among the 23 included studies, four studies had a high ROB, while seven demonstrated some concerns of ROB (Supporting Information 1: Figures S1 and S2). The major source of bias was the selection of the reported outcome, deviations from intended interventions, and the randomization process. The leave-one-out method suggested a moderate effect size and no noticeable influence while omitting a single study, thus, indicating good quality.

### 3.5. Publication Bias

The funnel plot based on depression symptoms is relatively symmetrical (Supporting Information 1: Figure S3), and the results of the Egger test are not statistically significant (*p*=0.47; Supporting Information 1: Table S2). Additionally, the trim-and-fill method and its adjusted funnel plot (Supporting Information 1: Figure S4) showed no hypothetical studies added or removed, suggesting no substantial publication bias when combined with the Egger test results. These results indicated that there is no significant publication bias in the current meta-analysis.

### 3.6. Sensitivity Analysis

**Figure 3:**
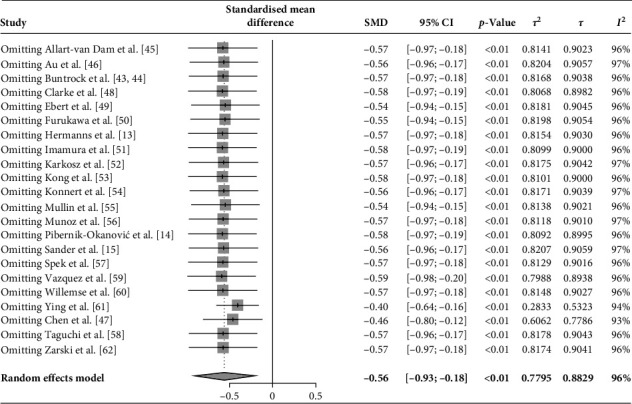
Sensitivity analysis by the leave-one-out method.

**Figure 4:**
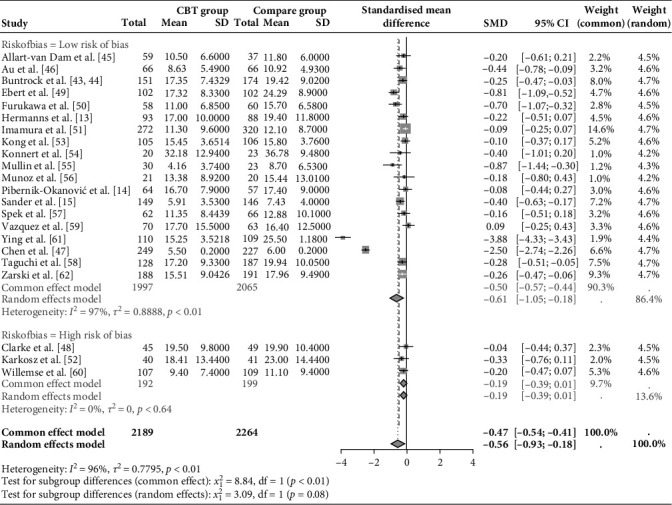
Sensitivity analysis based on the risk of bias. CI, confidence interval; SMD, standard mean difference.

The sensitivity analysis results shown in Figures 3 and 4 confirmed the stability of the study's findings. Excluding studies (*k* = 3) with high ROB slightly increased the SMD from −0.56 (95% CI: −0.93, −0.18) to −0.61 (95% CI: −1.05, −0.18), indicating a consistent treatment effect. Although there was a modest rise in heterogeneity, with *τ*^2^ increasing from 0.78 to 0.89, this change does not undermine the reliability of the meta-analysis. Additionally, omitting each study individually demonstrated that none of the included studies significantly influenced the pooled heterogeneity, indicating consistent contributions from all included studies.

**Figure 5:**
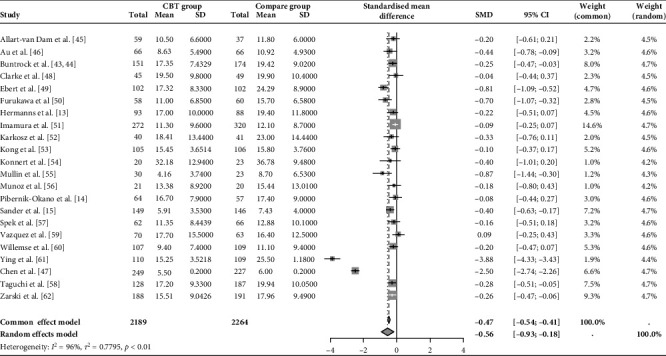
Changes in depressive symptoms during final follow-up assessment. CI, confidence interval; SMD, standard mean difference.

During the omitting method, results suggested that Ying et al. [61] and Chen et al. [47] might be potential outliers. Thus, the removal of Ying et al. [61], Chen et al. [47], and both studies was conducted individually (Supporting Information 1: Figures S5–S7). The results of separated SMD and 95% CI were reported as follows, respectively: −0.40 (95% CI: −0.64, −0.16); −0.46 (95% CI: −0.80, −0.12); −0.28 (95% CI: −0.38, −0.18). These results show reduced SMD and heterogeneity compared to the original results (Figure 5). The overall results were relatively robust to some extent after the exclusion of outliers, with no reversal of effect direction or change in statistical significance, supporting their retention in the pooled analysis. Therefore, all studies were retained in the final meta-analysis, reinforcing the robustness and stability of the findings.

### 3.7. Meta-Regression

To conduct the meta-regression, the variables (Supporting Information 1) were first identified according to a predefined methodology (Section 2.5).  Table 8 presents the results of the meta-regression analyses of the type of CBT approaches, the duration of the CBT in managing depressive symptoms, and the results of the meta-regression analyses of participant characteristics.

All variables were analyzed separately in the Univariate meta-regression model (intervention duration, number of CBT sessions, and the mean age of participants), results revealed that number of CBT sessions had a significant effect on study heterogeneity of subclinical depressive symptoms at follow-up and indicate the predictor's association with the results (number of CBT sessions: coefficient = −0.64, *p*=0.03).

In the multivariate meta-regression model, no variables with significant effects on study heterogeneity were found.

## 4. Discussion

The current systematic review and meta-analysis provide an overview and insight into the effectiveness of CBT, including the effectiveness of different CBT approaches that aim toward different targeted populations to comprehensively map the long-term effect of CBT in managing subclinical depression. In general, the current findings suggest that CBT is effective in managing subclinical depression. While the overall effect size was reduced from moderate to small after the removal of two statistical outliers [47, 61], the observed effect remains significant. This finding suggested that the main effect may be contributed by specific studies with extreme reported effects and must be interpreted with caution. In addition, it is important to note that the estimated RR can be biased if the sensitivity and specificity of the diagnostic instrument are not 100% precise [63]. Since most of the included studies in the current review relied on self-reported questionnaires for depression diagnosis, the RR estimates in the current review should be interpreted with caution, as potential misclassification bias may exist. Additionally, the current meta-analysis also aims to examine the effectiveness of CBT in managing anxiety symptoms and changes to the quality of life among the subclinical depression populations. Anxiety, as one of the most co-occurring is associated with negative and biased cognitive distortions, which in turn, lead to greater depression. The objective of CBT is to transfer negative thoughts or distortions into more adaptive thinking using different cognitive techniques [64]. Results from the current studies confirmed that the early implementation of CBT in the subclinical depression population would not only improve depression severity but also improve anxiety severity [29]. However, since the included studies also consisted of participants with chronic diseases, such as back pain and diabetes, the use of CBT might not effectively address the physical chronic condition that could bring disturbance to an individual's daily living, work, and social interaction, thus, might explain the lack of significant results regarding changes in quality of life. More studies are needed to further examine the change in quality of life using CBT among the subclinical depression population.

i-CBT emerges as the most effective form for managing subclinical depression, however, the moderate effectiveness of i-CBT may be attributed to barriers such as the requirement for computer skills and internet access [27], particularly among older adults. The flexibility of i-CBT also poses challenges, as participants may prematurely end or postpone treatment once symptoms improve [57], impacting its overall effectiveness.

While a significant effect was observed when interventions lasted less than 1 month, its effect is relatively weak, suggesting that short-term CBT may not be sufficient to yield measurable improvements in subclinical depressive symptoms overtime. This finding aligns with previous research indicating that CBT often requires time to fully realize its therapeutic effects [65]. However, the significantly lower efficacy observed in interventions lasting longer than 3 months compared to those lasting 1–3 months prompts speculation regarding potential drawbacks or a threshold effect associated with prolonged CBT interventions for subclinical depression. This hypothesis may stem from the possibility that participants in long-term interventions experience fatigue or reduced engagement, warranting further exploration.

The preventive effect of CBT diminishes after 12 months, highlighting the need for periodic booster sessions to sustain its effects. The remission and relapse characteristics of depression contribute to this diminishing effect, emphasizing the importance of long-term support and follow-up care in preventing relapse.

While CBT demonstrates slightly greater effectiveness in reducing depression symptoms compared to other nonpharmacological treatments, its effect size remains relatively small. It is important to note that there are only a limited number of studies that have implemented both CBT and other forms of nonpharmacological treatment in a comparable group. This suggests that while CBT holds promise as a first-line intervention for subclinical depression, caution is necessary in overestimating its efficacy. Future comparative research or multimodal intervention is warranted to determine whether CBT offers unique advantages over other interventions in the long-term management of subclinical depression.

The current study confirms previous research regarding the effectiveness of traditional individual-based face-to-face CBT and i-CBT in managing subclinical depression [66]. Due to the lack of studies that implemented traditional individual-based face-to-face CBT targeted at subclinical depression, its effectiveness remains unclear. However, g-CBT appears to be the least effective, potentially due to factors such as fear of stigmatization and the need for individual privacy [67]. Previous meta-analyses have consistently demonstrated that although group CBT may yield short-term benefits as an intervention for subclinical depression, its utility as a long-term preventive measure against the progression of subclinical depression to major depression is limited [68]. Despite its limited efficacy in the current findings, g-CBT may offer unique benefits through peer support and increased adherence if implemented carefully [69].

Gender differences and help-seeking behaviors significantly influence the effectiveness of CBT in managing subclinical depression. Attitudinal and structural barriers, particularly prevalent among males, ethnic minorities, and young adults, contribute to low help-seeking behavior, especially when seeking professional help might threaten their perceived masculinity, increase their fear of disclosure and stigmatization, and contradict their cultural beliefs [70]. It is important to note that depressive symptoms among those with subclinical depression are relatively mild when compared to those with major depression. Thereby, making people with subclinical depression less likely to seek professional help and keep depression within the family and a much harder-to-reach population either clinically or in research settings. This underscores the importance of considering demographic factors when designing and implementing CBT interventions.

Cultural beliefs and attitudes towards authority figures also play a crucial role in CBT effectiveness. Among publications from Asian countries, the large effect size might be attributed to the high adherence rates to CBT due to the cultural norms of obedience to authority. It is reported that the Chinese culture of obedience to authority toward doctors might play a role in the increasing adherence to CBT, thus, the use of intensive guidance from an authority figure such as therapists or medical professionals helped improve the adherence rate among the Chinese population [53, 61]. These cultural and contextual factors may explain the variations in treatment outcomes observed across different regions. Studies from Europe consistently yield stable and significant results, indicating the effectiveness of CBT in Western cultures. However, the small number of studies from Australia and North America warrant further investigation to validate these findings.

Age-related factors further complicate the effectiveness of CBT interventions. Older adults, particularly those with lower education levels, face challenges such as poor computer skills, leading to higher dropout rates in internet-based interventions [71]. This highlights the need for tailored interventions that accommodate the unique needs of diverse age groups. High attrition rates among older adults in non-pharmacological interventions emphasize the importance of implementing suitable forms of CBT tailored to specific populations.

In addition, although the effect of CBT on adults with subclinical depression is significant, the high heterogeneity across the studies caused by a variety of types of CBT approaches and intervention duration might play a role in the wide 95% CI interval. Besides, the current adult age range is rather wide (18–60 years old) based on the previous review [8]. Thus, the results must be interpreted with caution. More studies are warranted to further examine the effectiveness of CBT on adults with subclinical depression, particularly on young and middle-aged adults, to investigate the effectiveness of CBT on different stages of adulthood.

The current systematic review and meta-analysis provide evidence on the duration and type of intervention that could inform researchers and clinicians in future implementation of CBT for subclinical depression; however, the current meta-analysis has several limitations: (1) Since the proposed criteria for subclinical depression remain inconsistent among researchers, an update aligning with the revised diagnostic criteria of the ICD-11 should be made. This would allow future researchers to better focus and improve the mental health of the subclinical depression population. (2) Most findings rely on self-reported measures like the CES-D, which may lack accuracy and sensitivity, especially in populations with multiple chronic conditions [54]. Comorbidities such as diabetes and persistent pain might confound the results. (3) The majority of studies originated from Europe or Asia, limiting generalizability; future research should investigate CBT efficacy in North America and Australia, considering cultural variations. (4) Included studies employed diverse CBT interventions, with limited comparisons to other commonly used therapies, impeding direct effectiveness comparisons. It remains challenging to determine whether CBT outperforms alternative psychological interventions. (5) Definitions of educational levels varied across studies, precluding analysis of their impact on CBT effectiveness. This compromises the assessment of CBT implementation. (6) The study did not explore the relationship between CBT components and outcomes due to incomplete intervention descriptions in many studies, hindering the examination of component importance differences in CBT. (7) Given the small number of studies in some subgroup analyses, more studies are warranted for more stable and reliable results. (8) Given that many of the included studies relied on self-reported screening instruments rather than clinical diagnosis, the RR values and NNT may be influenced by the lack of professional diagnosis. This limitation aligns with Copeland et al. [63], who highlighted that the estimated RR can deviate from the true RR if classification is imprecise. Thus, the estimated RR and NNT could only act as a reference instead of strong evidence. Future studies on the use of CBT on subclinical depression should take the use of professional diagnosis during follow-up assessment into consideration to provide more robust evidence.

## 5. Conclusion

This systematic review and meta-analysis provide evidence suggesting that the use of i-CBT provided within one to 3 months with additional check-in or booster sessions at the 12-month follow-up might be the most effective way of managing depressive and anxiety symptoms among the subclinical depression population. Although the results of the current study indicated the preventive effects of CBT in the transition from subclinical depression into clinical depression or major depression, results were limited by self-reported measurement and must be interpreted with caution.

Nevertheless, the current study supports CBT as a first-line intervention for subclinical depression. It also provides further insight into its application, emphasizing the need for tailored CBT approaches for specific populations and highlighting the importance of reminders and guidance to enhance implementation success.

## Figures and Tables

**Table 1:** Study and participants characteristic of the included studies.

References	Country	Sample size	Age (mean ± SD)	Gender (male/female)	Comorbid disease	Type of CBT	Type of control	Intervention duration	Intervention frequency	Follow-up time point
[45]	Netherland	110	45.6 ± 9.9	44/66	—	g-CBT	TAU	12 wk	Once per wk	6, 12 months

[46]	Hong Kong	132	<70	—	Type 2 diabetes mellitus	g-CBT	TAU	3 months	Once per wk	3 months

[43]	Germany	406	45 ± 11.9	106/300	—	i-CBT	Internet-PsyE	6 wk	Once or twice per wk	6 months

[44]	Germany	406	45 ± 11.9	106/300	—	i-CBT	Internet-PsyE	6 wk	Once or twice per wk	12 months

[47]	China and Hong Kong	708	i-CBT: 21.9 ± 2 HE: 22.2 ± 1.9	i-CBT: 152/202 HE: 149/205	Insomnia	i-CBT	HE	6 wk	Once per wk	6, 12 months

[48]	United States of America	87	g-CBT: 14.4 ± 1.4 TAU: 14.7 ± 1.5	g-CBT: 16/24 TAU: 15/32	—	g-CBT	TAU	—	15 sessions	12, 24 months
[49]	Germany	204	44.2 ± 11.7	40/164	—	i-CBT	WL	7 wk	Once or twice per wk	3 months

[50]	Japan	118	—	92/26	—	t-CBT + EAP	WL + EAP	4 months	8 sessions	8 months

[13]	Germany	214	43.3 ± 13.3	93/121	Type 2 diabetes	g-CBT	HE	—	5 sessions	6, 12 months

[51]	Japan	762	i-CBT: 38 ± 9.2 HE: 37.2 ± 8.8	iCBT: 325/56 HE: 314/67	—	i-CBT	HE	6 wk	Once per wk	3, 6 months

[52]	Poland	81	i-CBT: 26.6 ± 5.1 PsyE: 24.8 ± 4.0	i-CBT:11/29 PsyE: 29/10	—	i-CBT	PsyE	2 wk	—	1 month

[53]	China	315	71.6 ± 7.8	100/215	—	1. i-CBT 2. g-CBT	WL	5 wk	Once per wk	6, 12 months

[54]	Canada	43	81.1 ± 6.8	10/33	—	g-CBT	TAU	7 wk	Twice per wk	3, 6 months

[55]	Australia	53	i-CBT: 28.6 ± 10.1 WL: 26.9 ± 11.5	iCBT: 11/19 WL: 8/15	Experience anxiety and depression	i-CBT	WL	17 wk	—	3 months

[56]	United States of America	41	g-CBT: 24.8 ± 4.2 TAU: 25 ± 4.7	g-CBT: 0/21 TAU: 0/20	Postpartum women	g-CBT	TAU	12 wk	Once per wk	3, 6, 12 months

[14]	Croatia	209	g-CBT: 57.7 ± 6.2 PE: 58.5 ± 4.8 EUC: 58.2 ± 5.6	g-CBT: 34/40 PE: 29/37 EUC: 33/36	Type 2 diabetes	g-CBT	1. PE 2. EUC	6 wk	Once per wk	6, 12 months

[15]	Germany	295	i-CBT: 51.7 ± 8.5 TAU: 53.9 ± 6.7	i-CBT: 60/89 TAU: 51/95	—	i-CBT	TAU	9 wk	6 session and 3 optional modules	6, 12 months

[57]	Netherland	292	i-CBT: 55 ± 4.9 g-CBT: 54 ± 3.9 WL: 55 ± 5	i-CBT: 33/69 g-CBT: 36/63 WL: 41/59	—	1. i-CBT 2. g-CBT	WL	8 wk 10 wk	Once per wk	—

[42]	Netherland	70	in-CBT: 18.62 (4.27) CG: 17.69 (4.82)	22/48	Inflammatory bowel disease (IBD)	In-CBT	regular medical consultations	3 months	Once per wk	6, 12 months

[58]	Japan	846	i-CBT: 41.3 ± 12.5 Sham: 42.5 ± 11.7	i-CBT: 130/294 Sham: 130/292	—	i-CBT	Sham Treatment	4 wk	—	1 month

[59]	Spain	133	g-CBT: 23.9 ± 5.4 RT: 22.5 ± 3.1	g-CBT: 14/56 RT: 10/53	—	g-CBT	RT	8 wk	Once per wk	3, 6 months

[60]	Netherland	216	in-CBT: 39.4 ± 11.4 TAU: 41.8 ± 11.2	in-CBT: 36/71 TAU: 37/72	—	in-CBT	TAU	14 wk	—	12 months

[61]	China	268	i-CBT: 39.9 ± 13.5 in-CBT: 43.7 ± 14.2 WL: 40.4 ± 12.1	i-CBT: 31/60 in-CBT: 28/57 WL: 31/61	—	1. i-CBT 2. in-CBT	WL	5 wk	Once per wk	6 months

[62]	Germany and Switzerland	566	in-CBT: 40.6 ± 14.3 i-CBT: 38.7 ± 13.7 WL: 41.1 ± 14.0	in-CBT: 46/140 i-CBT: 55/134 WL: 55/136	Subclinical anxiety	1. in-CBT 2. i-CBT	WL	—	—	6, 12 months

Abbreviations: CBT, cognitive behavioral therapy; EUC, enhanced usual care; g-CBT, group cognitive behavioral therapy; HE, health education; i-CBT, internet cognitive behavioral therapy; i-PST, internet problem-solving therapy; in-CBT, individual cognitive behavioral therapy; PE, physical exercise; PsyE, psychoeducation; RT, relaxation training; t-CBT, telephone cognitive behavioral therapy; TAU, treatment as usual; wk, weeks; WL, waitlist.

**Table 2:** Subgroup analyses based on participant and study characteristics.

Characteristic	Number of studies	Meta-analysis		Heterogeneity		Between-group test	
		Hedges's *g*	95% CI	*I* ^2^	*p*	Chi^2^	*p*
Participant characteristics							
Mean age							
Children and adolescents	1	—	—	—	—	1.78	0.18
Adults (18–60 years old)	18	−0.63	−1.08 to −0.17^a^	97%	<0.01		
Older adults (above 60 years old)	3	−0.27	−0.53 to −0.00^a^	24%	0.27		
Gender							
Male < female	18	−0.59	−1.05 to −0.13^a^	97%	<0.01	0.71	0.70
Male > female	3	−0.34	−0.71 to 0.03	78%	0.01		
No report	1	−0.44	−0.78 to −0.09^a^	—	—		
Associated disease							
With disease	7	−0.22	−0.36 to −0.08^a^	26%	0.23	2.91	0.09
No mention disease	15	−0.71	−1.25 to −0.17^a^	97%	<0.01		
Study characteristics							
Year of publication							
10 years ago	8	−0.21	−0.39 to −0.03^a^	35%	0.15	3.08	0.08
Recent 10 years	14	−0.74	−1.31 to −0.18^a^	98%	<0.01		
Country of publication							
Asia	7	−1.13	−2.22 to −0.04^a^	99%	<0.01	7.19	0.07
Australia	1	−0.87	−1.44 to −0.30^a^	—	—		
Europe	11	−0.27	−0.40 to −0.14^a^	53%	0.02		
North America	3	−0.16	−0.45 to 0.14	0%	0.62		
Types of CBT approaches							
in-CBT	2	−2.87	−8.18 to 2.44	—	—	7.88	0.05
g-CBT	10	−0.11	−0.24 to 0.02	9%	0.36		
i-CBT	12	−0.81	−1.38 to −0.25^a^	98%	<0.01		
t-CBT	2	−0.43	−0.91 to 0.05	—	—		
Intervention duration							
<1 M	3	−0.24	−0.42 to −0.06^a^	0%	0.55	2.13	0.34
1 –3 M	14	−0.69	−1.27 to −0.11^a^	98%	<0.01		
>3 M	5	−0.31	−0.45 to −0.16^a^	23%	0.27		
Follow-up duration							
FU < 6 M	4	−0.69	−0.90 to −0.48^a^	18%	0.30	1.85	0.40
6 ≤ FU < 12 M	6	−0.83	−2.03 to 0.37	98%	<0.01		
FU ≥ 12 M	12	−0.39	−0.78 to 0.00	96%	<0.01		
Attrition rate							
High	9	−0.98	−1.83 to −0.12^a^	98%	<0.01	2.83	0.09
Low and moderate	13	−0.24	−0.36 to −0.12^a^	48%	0.03		

*Note:* Justification for each subgroup type: age, characterized into three groups: children and adolescents (0–17), adults (18–60), and older adults (above 60) based on previous cutoff [8]; gender: male < female and based on previous review suggested that females are three times more likely to have subclinical depression [8]; associated disease: to see if the occurring symptoms from other diseases would influence the intervention outcome. Intervention duration, characterized into three groups: <1 M, 1–3 M, >3 M to ensure there are enough studies to conduct subgroup analysis; follow-up duration, characterized into three groups: 1 < FU ≤ 6 M, 6 < FU < 12, and ≥12 M due to the lack of studies conducted follow-up in less than 3 months, in 3 months, and 6 month which might result in low statistical power; attrition rate, characterized into two groups: high (≥30%) and low and moderate (<30%), the merge of low and moderate attrition was due to the lack of studies that have <10% attrition which might result in low statistical power.

^a^Represents statistically significant.

**Table 3:** Comparison between CBT and other forms of treatment.

Study	Type of CBT	Type of control	Hedges's *g*	95% CI	Major finding
Buntrock et al. [43, 44]	i-CBT	Internet psychoeducation	−0.28	−0.50 to −0.06	Greater significant improvement in depressive symptoms was found in the i-CBT group compared to the control group
Pibernik-Okanović et al. [14]	g-CBT	1. Physical exercise 2. EUC	−0.16	−0.51 to 0.20	There was a significant improvement in depressive symptoms, but no significant difference between all groups
Vázquez et al. [59]	g-CBT	Relaxation training	0.09	−0.25 to 0.43	There was a significant improvement in depressive symptoms, but no significant difference between all groups
Overall: *I*^2^ = 39%; *g* = −0.14, Sig (*p*=0.20)					−0.14 (−0.37 to 0.08)

Abbreviations: CBT, cognitive behavioral therapy; CI, confidence interval; EUC, enhanced usual care; g-CBT, group cognitive behavioral therapy; i-CBT, internet cognitive behavioral therapy; i-PST, internet problem-solving therapy; NS, no significant difference; WL, waitlist.

**Table 4:** Comparison between internet cognitive behavioral therapy and other types of CBT approach.

Study	Experimental group	Comparison group	Hedges's *g*	95% CI	Major finding
Kong et al. [53]	i-CBT	g-CBT	−0.48	−0.75 to −0.20	Greater significant improvement in depressive symptoms in the i-CBT group compared to the g-CBT group
Spek et al. [57]	i-CBT	g-CBT	−0.20	−0.55 to 0.15	There was a significant improvement in depressive symptoms, but no significant difference between the i-CBT and g-CBT groups
Ying et al. [61]	i-CBT	in-CBT	−0.26	−0.52 to 0.01	Greater significant improvement in depressive symptoms in the i-CBT group compared to the in-CBT group
Zarski et al. [62]	i-CBT	in-CBT	−0.18	−0.38 to 0.02	There was a significant improvement in depressive symptoms, but no significant difference between the i-CBT and in-CBT groups
Overall: *I*^2^ = 4%, *g* = −0.27, Sig (*p*=0.37)					−0.27 (−0.41 to −0.13)

Abbreviations: CBT, cognitive behavioral therapy; CI, confidence interval; g-CBT, group cognitive behavioral therapy; i-CBT, internet cognitive behavioral therapy; in-CBT, individual cognitive behavioral therapy; NS, no significant difference; TAU, treatment as usual; WL, waitlist.

**Table 5:** Risk ratio of the experimental and control groups at the follow-up stage.

Study	Follow-up duration	Experimental group		Comparison group		RR	95% CI	Major findings
		Events.M	Total	Events.M	Total			
[45]	12 month	19	68	11	42	1.07	0.57–2.01	No evidence was found that the coping with depression course prevented depressive episodes
[47]	12 month	37	354	62	354	0.60	0.41–0.87	This result indicates that the risk of developing major depression disorder was significantly lower in the i-CBT group when compared to the control group
[48]	24 month	8	40	16	47	0.59	0.28–1.23	This significant preventive effect persisted, but at a diminished level at the 18- and 24-month follow-ups
[13]	12 month	10	93	20	88	0.47	0.23–0.95	This result indicates that effective treatment of subclinical depression may yield a preventive effect for the exacerbation of depressive symptoms
[54]	6 month	0	20	2	23	0.23	0.01–4.50	The results of this study suggest that CBT is beneficial for residents with subclinical depression
[56]	12 month	0	21	2	20	0.19	0.01–3.75	This study found that the intervention group had a lower MDE incidence than the comparison group, although this difference did not attain statistical significance
[15]	12 month	21	149	41	146	0.50	0.31–0.81	This result demonstrated that, for patients with subclinical depressive symptoms and persistent back pain, intervention significantly reduced the incidence of MDE over a 12-month follow-up period
[42]	12 month	2	36	1	32	1.78	0.17–18.69	The groups did not differ in the proportion of patients that developed clinical anxiety and/or depression
[59]	6 month	6	65	5	59	1.09	0.35–3.38	In fact, except for a very small number of indicated prevention studies in which a decrease in the incidence of depression was found, most studies have only been able to detect reductions in depression symptoms
[60]	12 month	13	107	20	109	0.66	0.35–1.26	The incidence of major depression among participants in the experimental condition was reduced by a third compared with the control condition
Overall: *I*^2^ = 0%, RR = 0.62, Sig (*p*=0.64) Total RR = 0.62; 95% CI (0.50–0.77)								NNT = 8.79; 95% CI: 6.67–14.55

Abbreviations: CBT, cognitive behavioral therapy; CI, confidence interval; MDE, major depressive episode; NNT, number needed to treat; RR, risk ratio.

**Table 6:** Changes in anxiety symptoms.

Study	Type of CBT approach	Hedges's *g* (95% CI)
Postassessment		
Buntrock et al. [43]	i-CBT	−0.52 (−0.72 to −0.32)
Chen et al. [47]	i-CBT	−4.49 (−4.80 to −4.19)
Ebert et al. [49]	i-CBT	−0.22 (−0.49 to 0.06)
Karkosz et al. [52]	i-CBT	0.03 (−0.41 to 0.47)
Kong et al. [53]	i-CBT and g-CBT	−0.75 (−1.03 to −0.47)
Mullin et al. [55]	i-CBT	−0.47 (−1.02 to 0.08)
Vázquez et al. [59]	g-CBT	0.31 (−0.03 to 0.65)
Taguchi et al. [58]	i-CBT	−0.08 (−0.30 to 0.15)
Ying et al. [61]	i-CBT and in-CBT	−2.60 (−2.96 to –2.24)
Zarski et al. [62]	i-CBT and in-CBT	−0.43 (−0.63 to −0.23)
Overall: *I*^2^ = 99%; *g* = −0.92, Sig (*p* < 0.01)		−0.92 (−1.84 to −0.00)
At follow-up		
Buntrock et al. [44]	i-CBT	−0.38 (−0.60 to −0.16)
Chen et al. [47]	i-CBT	−2.00 (−2.22 to −1.78)
Ebert et al. [49]	i-CBT	−0.44 (−0.72 to −0.16)
Karkosz et al. [52]	i-CBT	−0.23 (−0.67 to 0.20)
Kong et al. [53]	i-CBT and g-CBT	−0.98 (−1.27 to −0.70)
Mullin et al. [55]	i-CBT	−0.87 (−1.44 to −0.30)
Vázquez et al. [59]	g-CBT	−0.08 (−0.42 to 0.26)
Taguchi et al. [58]	i-CBT	−0.17 (−0.39 to 0.06)
Ying et al. [61]	i-CBT and in-CBT	−2.58(−2.94 to –2.22)
Zarski et al. [62]	i-CBT and in-CBT	−0.32 (−0.52 to −0.12)
Overall: *I*^2^ = 97%; *g* = −0.70, Sig (*p* < 0.01)		−0.70 (−1.15 to −0.25)

Abbreviations: CI, confidence interval; g-CBT, group cognitive behavioral therapy; i-CBT, internet cognitive behavioral therapy; in-CBT, individual cognitive behavioral therapy.

**Table 7:** Changes in quality of life.

Study	Type of CBT approach	Hedges's *g* (95% CI)
Hermanns et al. [13]	g-CBT	−0.05 (−0.34 to 0.25)
Pibernik-Okanović et al. [14]	g-CBT	0.18 (−0.17 to 0.54)
Sander et al. [15]	i-CBT	−0.22 (−0.45 to 0.01)
Overall: *I*^2^ = 44%; *g* = −0.06 NS (*p*=0.17)	—	−0.06 (−0.28 to 0.16)

Abbreviations: CI, confidence interval; g-CBT, group cognitive behavioral therapy; i-CBT, internet cognitive behavioral therapy.

**Table 8:** Results of exploratory post hoc meta-regression analyses.

Continuous variables	Multivariate regression model					Univariate regression model				
	Coefficient	SE	*p*	95% CI		Coefficient	SE	*p*	95% CI	
Mean age	−0.04	0.71	0.96	−1.60	1.53	0.32	0.37	0.40	−0.45	1.09
Intervention duration	0.11	0.34	0.74	−0.63	0.86	−0.04	0.50	0.94	−1.08	1.00
Numbers of CBT sessions	0.00	0.02	0.83	−0.04	0.05	−0.64	0.27	**0.03**	−1.21	−0.07

*Note:* Bold indicates statistically significant.

Abbreviations: CI, confidence interval; SE, standard error.

## Data Availability

The data that support the findings of this study are available from the corresponding author upon request.

## Supporting Information

Supporting Information 1 All the additional results of data analysis, figures, and tables mentioned in the current systematic review and meta-analysis were displayed in the supporting information.

Supporting Information 2 The PRISMA 2020 checklist for systematic review was displayed in the supporting information.
