# Supplementary material for: Long-Term Effect of Cognitive Behavioral Therapy in Managing Subclinical Depression: A Systematic Review and Meta-Analysis
Source: Depress Anxiety. 2025 Aug 15;2025:1610909. doi: 10.1155/da/1610909 (PMC12373477; doi:10.1155/da/1610909)
Supplement: Supporting Information 1 — All the additional results of data analysis, figures, and tables mentioned in the current systematic review and meta-analysis were displayed in the supporting information. [file 1610909.f1.docx]

| **Supplementary Table 1.** Components of CBT used in the included studies. | | | | | | |
| --- | --- | --- | --- | --- | --- | --- |
| ***Study*** | **Psychoeducation** | **Identifying negative thoughts** | **Cognitive reconstruction** | **Homework** | **Relapse prevention** | **Self-monitoring** |
| Allart-van Dam et al., 2007 | ● | ● | ● | ● | ● | ● |
| Au et al., 2022 | ● | ● | ● |  | ● | ● |
| Buntrock et al., 2015 |  | ● | ● | ● |  |  |
| Buntrock et al., 2016 |  | ● | ● | ● |  |  |
| Chen et al., 2025 | ● | ● | ● | ● | ● | ● |
| Clarke et al., 2001 |  | ● | ● | ● |  |  |
| Ebert et al., 2018 |  | ● | ● | ● |  |  |
| Furukawa et al., 2012 | ● | ● | ● | ● |  | ● |
| Hermanns et al., 2015 | ● | ● | ● | ● | ● |  |
| Imamura et al., 2014 | ● | ● | ● | ● |  | ● |
| Karkosz et al., 2024 | ● | ● | ● | ● |  |  |
| Kong et al., 2024 |  |  |  |  |  |  |
| Konnert et al., 2009 | ● | ● | ● | ● | ● |  |
| Mullin et al., 2015 | ● | ● | ● |  | ● |  |
| Muñoz et al., 2007 | ● | ● | ● | ● |  | ● |
| Okanović et al., 2015 | ● | ● | ● | ● |  |  |
| Sander et al., 2020 | ● | ● | ● | ● |  |  |
| Spek et al., 2007 | ● | ● | ● |  | ● |  |
| Taguchi et al., 2025 | ● | ● | ● |  | ● | ● |
| Vázquez et al., 2012 | ● | ● | ● | ● |  |  |
| Willemse et al., 2004 | ● |  |  | ● |  |  |
| Ying et al., 2023 | ● | ● | ● |  | ● |  |
| Zarski et al., 2024 | ● | ● | ● |  | ● | ● |
| Note. Not all studies provide detailed information regarding the CBT components used in their study. | | | | | | |

| **Supplementary Table 2.** Egger Regression Test for the Assessment of Publication Bias | | | |
| --- | --- | --- | --- |
| **Bias estimate** | | **Test result** | |
| **SMD** | **SE** | ***t*** | ***p*** |
| -2.48 | 3.40 | -0.73 | 0.47 |
| Note. SE = standard error; SMD = standard mean difference. | | | |

**Supplementary- Keywords Used in Search of Studies**

For **CINAHL** (35):

AB ( cognitive-behavioral therapy OR cognitive-behavioural therapy OR cognitive-behavioral treatment OR cognitive-behavioural treatment OR CBT ) AND AB ( subclinical depression OR early-onset depression OR subsyndromal depression OR subthreshold depression )

For **Embase** (66):

('cognitive-behavioral therapy':ab,ti OR 'cognitive-behavioural therapy':ab,ti OR 'cognitive-behavioral treatment':ab,ti OR 'cognitive-behavioural treatment':ab,ti OR cbt:ab,ti) AND ('subclinical depression':ab,ti OR 'early-onset depression':ab,ti OR 'subsyndromal depression':ab,ti OR 'subthreshold depression':ab,ti)

For **Medline** (74):

AB ( cognitive-behavioral therapy OR cognitive-behavioural therapy OR cognitive-behavioral treatment OR cognitive-behavioural treatment OR CBT ) AND AB ( subclinical depression OR early-onset depression OR subsyndromal depression OR subthreshold depression )

For **PubMed** (60):

((cognitive-behavioral therapy[Title/Abstract] OR cognitive-behavioural therapy[Title/Abstract] OR cognitive-behavioral treatment[Title/Abstract] OR cognitive-behavioural treatment[Title/Abstract] OR CBT[Title/Abstract])) AND ((subclinical depression[Title/Abstract] OR early-onset depression[Title/Abstract] OR subsyndromal depression[Title/Abstract] OR subthreshold depression[Title/Abstract]))

For **PsycINFO** (160):

tiab(cognitive-behavioral therapy OR cognitive-behavioural therapy OR cognitive-behavioral treatment OR cognitive-behavioural treatment OR cbt) AND tiab(subclinical depression OR early-onset depression OR subsyndromal depression OR subthreshold depression)

For **Scopus** (93):

( TITLE-ABS-KEY ( cognitive-behavioral  AND  therapy  OR  cognitive-behavioural  AND  therapy  OR  cognitive-behavioral  AND  treatment  OR  cognitive-behavioural  AND  treatment  OR  cbt )  AND  TITLE-ABS-KEY ( subclinical  AND  depression  OR  early-onset  AND  depression  OR  subsyndromal  AND  depression  OR  subthreshold  AND  depression ) )  AND  ( LIMIT-TO ( EXACTKEYWORD ,  "Human" ) )

For **Web of Science** (185):

(AB=(cognitive-behavioral therapy OR cognitive-behavioural therapy OR cognitive-behavioral treatment OR cognitive-behavioural treatment OR CBT)) AND AB=(subclinical depression OR early-onset depression OR subsyndromal depression OR subthreshold depression)

**Supplementary- Keywords Used in Search of Studies (Updated Search On March 17 2024)**

For **CINAHL** (0):

AB ( cognitive-behavioral therapy OR cognitive-behavioural therapy OR cognitive-behavioral treatment OR cognitive-behavioural treatment OR CBT ) AND AB ( subclinical depression OR early-onset depression OR subsyndromal depression OR subthreshold depression )

For **Embase** (3):

('cognitive-behavioral therapy':ab,ti OR 'cognitive-behavioural therapy':ab,ti OR 'cognitive-behavioral treatment':ab,ti OR 'cognitive-behavioural treatment':ab,ti OR cbt:ab,ti) AND ('subclinical depression':ab,ti OR 'early-onset depression':ab,ti OR 'subsyndromal depression':ab,ti OR 'subthreshold depression':ab,ti)

For **Medline** (4):

AB ( cognitive-behavioral therapy OR cognitive-behavioural therapy OR cognitive-behavioral treatment OR cognitive-behavioural treatment OR CBT ) AND AB ( subclinical depression OR early-onset depression OR subsyndromal depression OR subthreshold depression )

For **PubMed** (4):

((cognitive-behavioral therapy[Title/Abstract] OR cognitive-behavioural therapy[Title/Abstract] OR cognitive-behavioral treatment[Title/Abstract] OR cognitive-behavioural treatment[Title/Abstract] OR CBT[Title/Abstract])) AND ((subclinical depression[Title/Abstract] OR early-onset depression[Title/Abstract] OR subsyndromal depression[Title/Abstract] OR subthreshold depression[Title/Abstract]))

For **PsycINFO** (7):

tiab(cognitive-behavioral therapy OR cognitive-behavioural therapy OR cognitive-behavioral treatment OR cognitive-behavioural treatment OR cbt) AND tiab(subclinical depression OR early-onset depression OR subsyndromal depression OR subthreshold depression)

For **Scopus** (10):

( TITLE-ABS-KEY ( cognitive-behavioral  AND  therapy  OR  cognitive-behavioural  AND  therapy  OR  cognitive-behavioral  AND  treatment  OR  cognitive-behavioural  AND  treatment  OR  cbt )  AND  TITLE-ABS-KEY ( subclinical  AND  depression  OR  early-onset  AND  depression  OR  subsyndromal  AND  depression  OR  subthreshold  AND  depression ) )  AND  ( LIMIT-TO ( EXACTKEYWORD ,  "Human" ) )

For **Web of Science** (12):

(AB=(cognitive-behavioral therapy OR cognitive-behavioural therapy OR cognitive-behavioral treatment OR cognitive-behavioural treatment OR CBT)) AND AB=(subclinical depression OR early-onset depression OR subsyndromal depression OR subthreshold depression)

**Supplementary-Inclusion and Exclusion Criteria**

***Inclusion criteria***

Participants: Must be defined as having subclinical depression explicitly by either self-report measurement on a well-developed depression scale or by fulfilling the criteria proposed by Judd et al. (1994) without the presence of major depressive disorder categorized by the DSM-5 or ICD-10. There were no restrictions on age, sex, and ethnicity.

Intervention: Must be conducted using cognitive-behavioral therapy, either by individual-based or group-based face-to-face cognitive behavioral therapy (in-CBT and g-CBT, respectively), internet-based CBT (i-CBT), or telephone-based CBT (t-CBT).

Comparator: Must include active control (other forms of CBT, other forms of therapy or treatment) or inactive control (treatment as usual or waitlist).

Outcomes: Changes in symptoms assessed by standardized and well-developed depression, anxiety, and quality of life scale, and must contain follow-up at least one month after the intervention.

Study Design: Must be randomized clinical trials (RCTs) published in English.

***Exclusion criteria***

Studies were excluded as follows: (1) were duplicate studies, (2) did not contain follow-up, (3) were not published in English, (4) were no RCT, (5) did not report original data, including studies that were systematic reviews, meta-analyses, or protocols, (6) did not conduct any forms of CBT, (7) did not report the outcomes of interest (change in depressive symptoms), and (8) did not contain relevant populations.

| **Supplementary – Figure 1.** Traffic Light Plot for Risk of Bias of the Included Studies |
| --- |
| 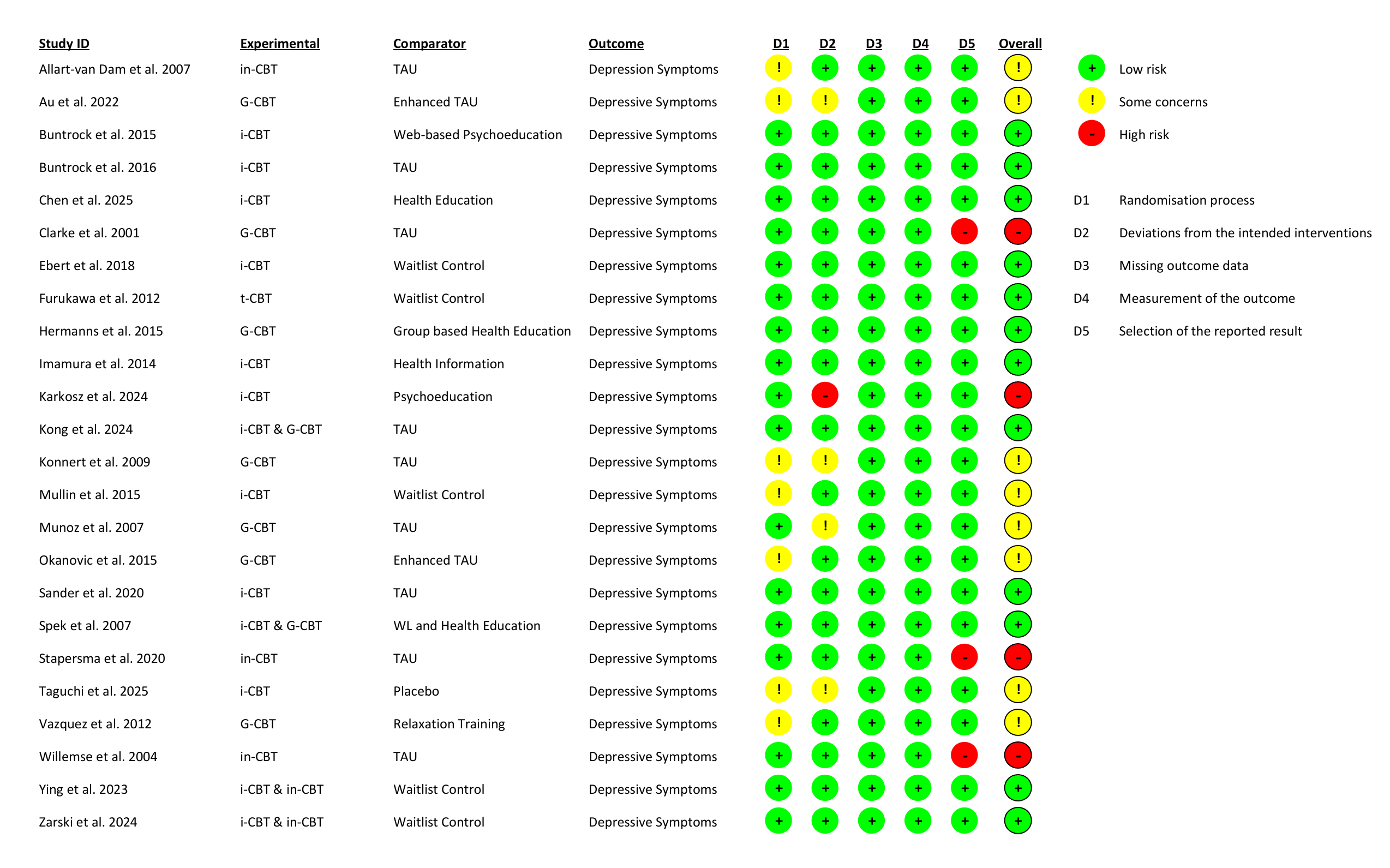 |
|  |

| **Supplementary – Figure 2.** The Overall Risk of Bias Summary |
| --- |
| 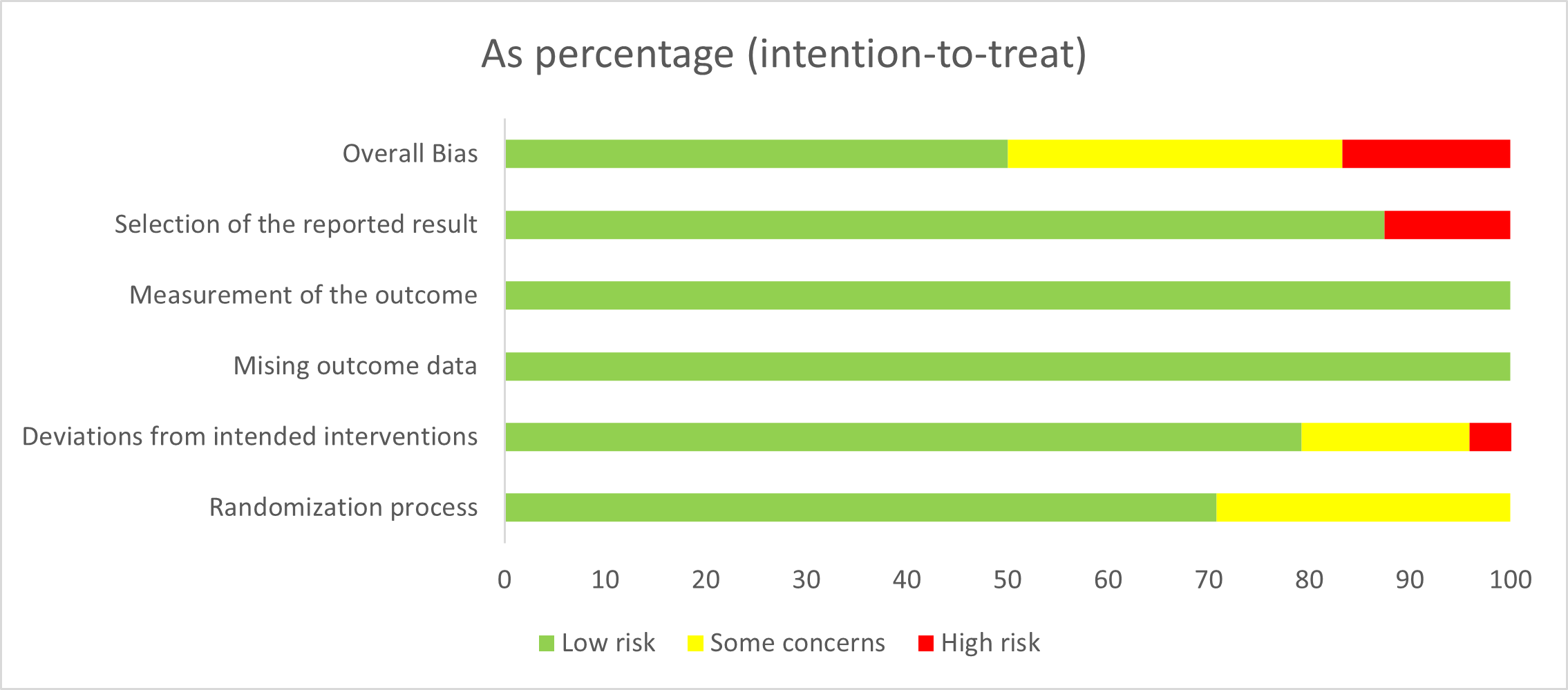 |

| **Supplementary – Figure 3.** Funnel Plot Assessing Publication Bias among the included studies (Depression symptom) |
| --- |
| 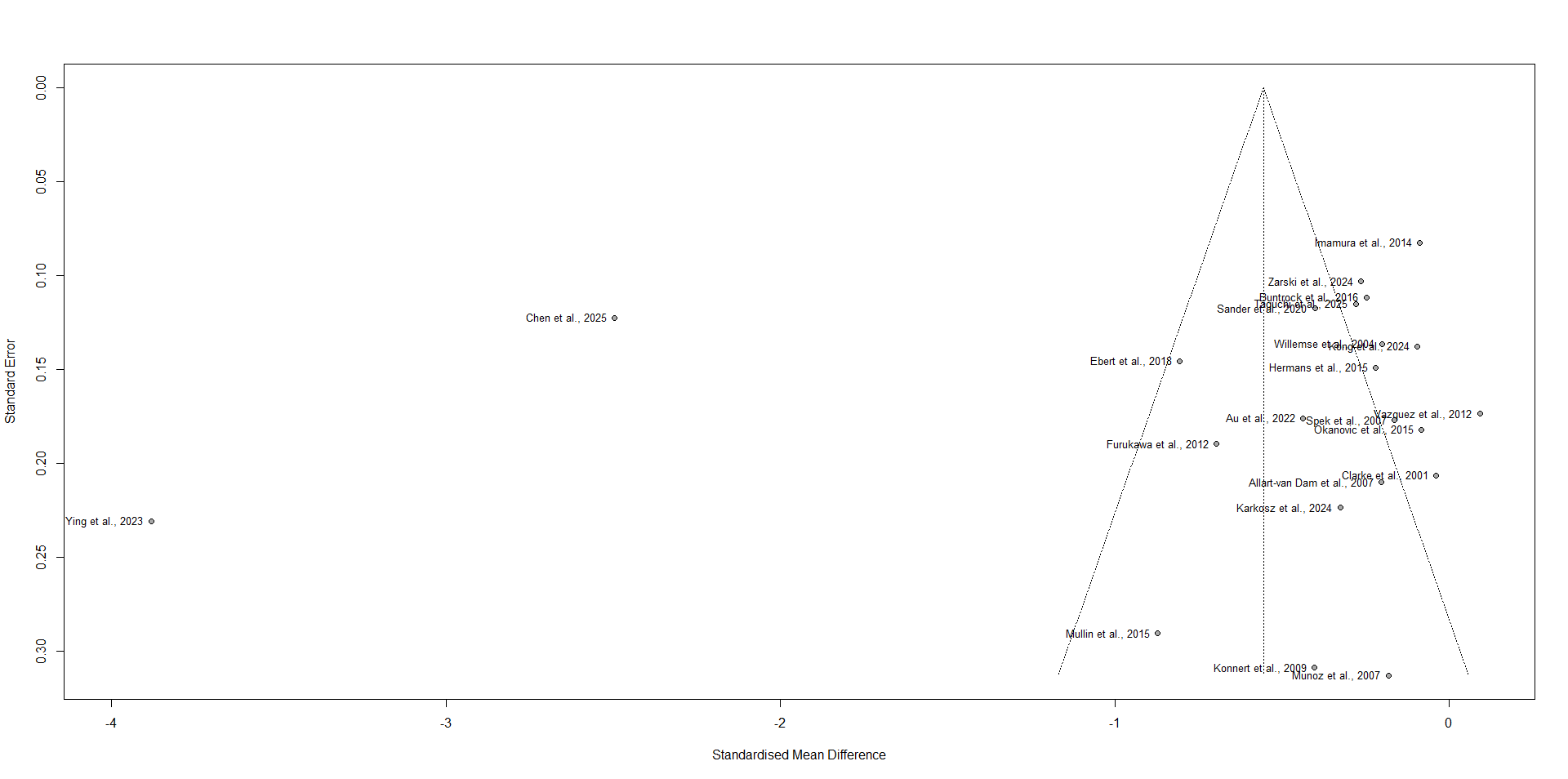 |
|  |

| **Supplementary – Figure 4.** Funnel Plot Assessing Publication Bias among the included studies after Trim-and-Fill method (Depression symptom) |
| --- |
| **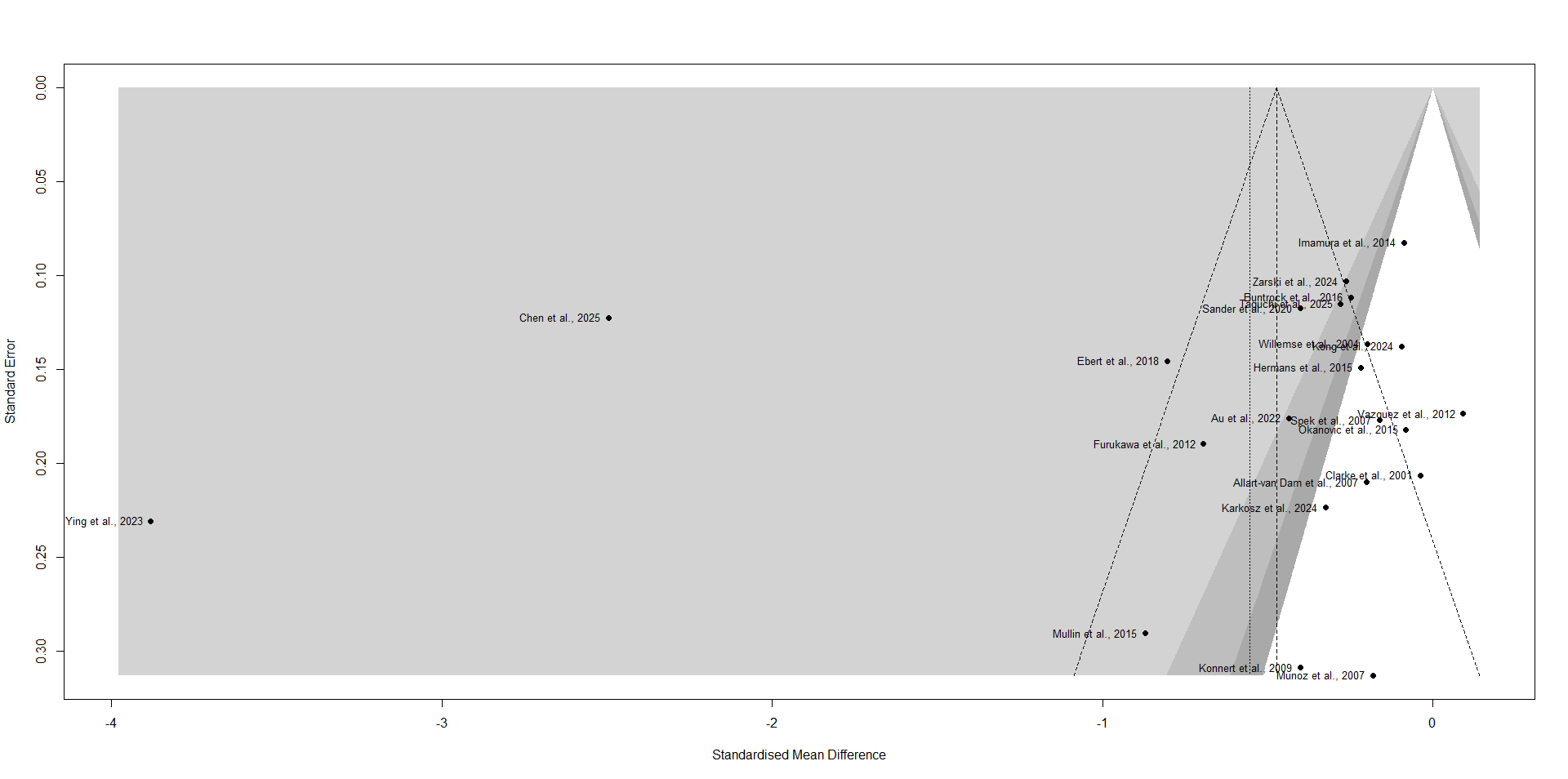** |

| **Supplementary – Figure 5.** Sensitive Analysis After the removal of Ying et al. 2023 |
| --- |
| **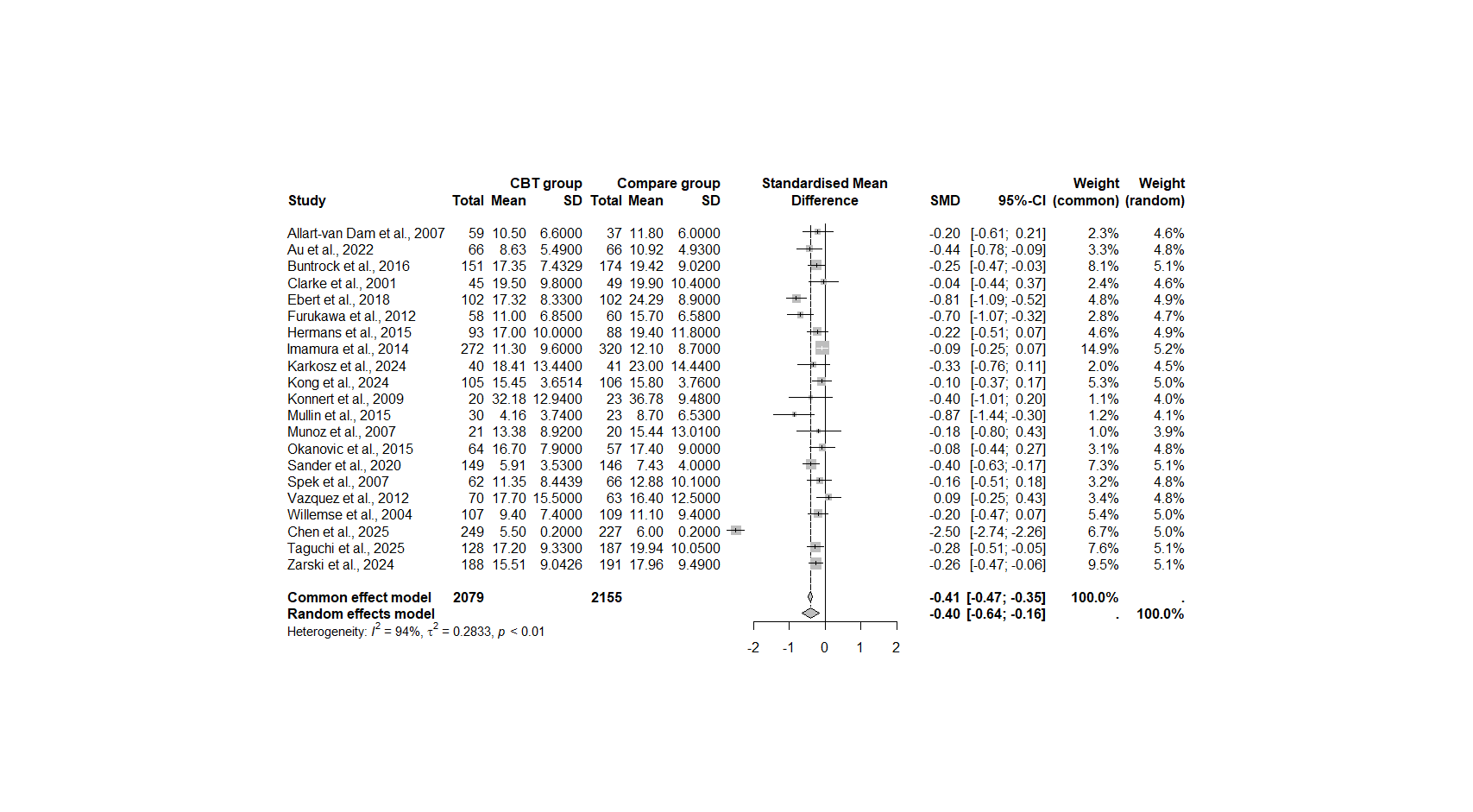** |
| Note. CI = confident interval; SMD = standard mean difference. |

| **Supplementary – Figure 6.** Sensitive Analysis After the removal of Chen et al. 2025 |
| --- |
| **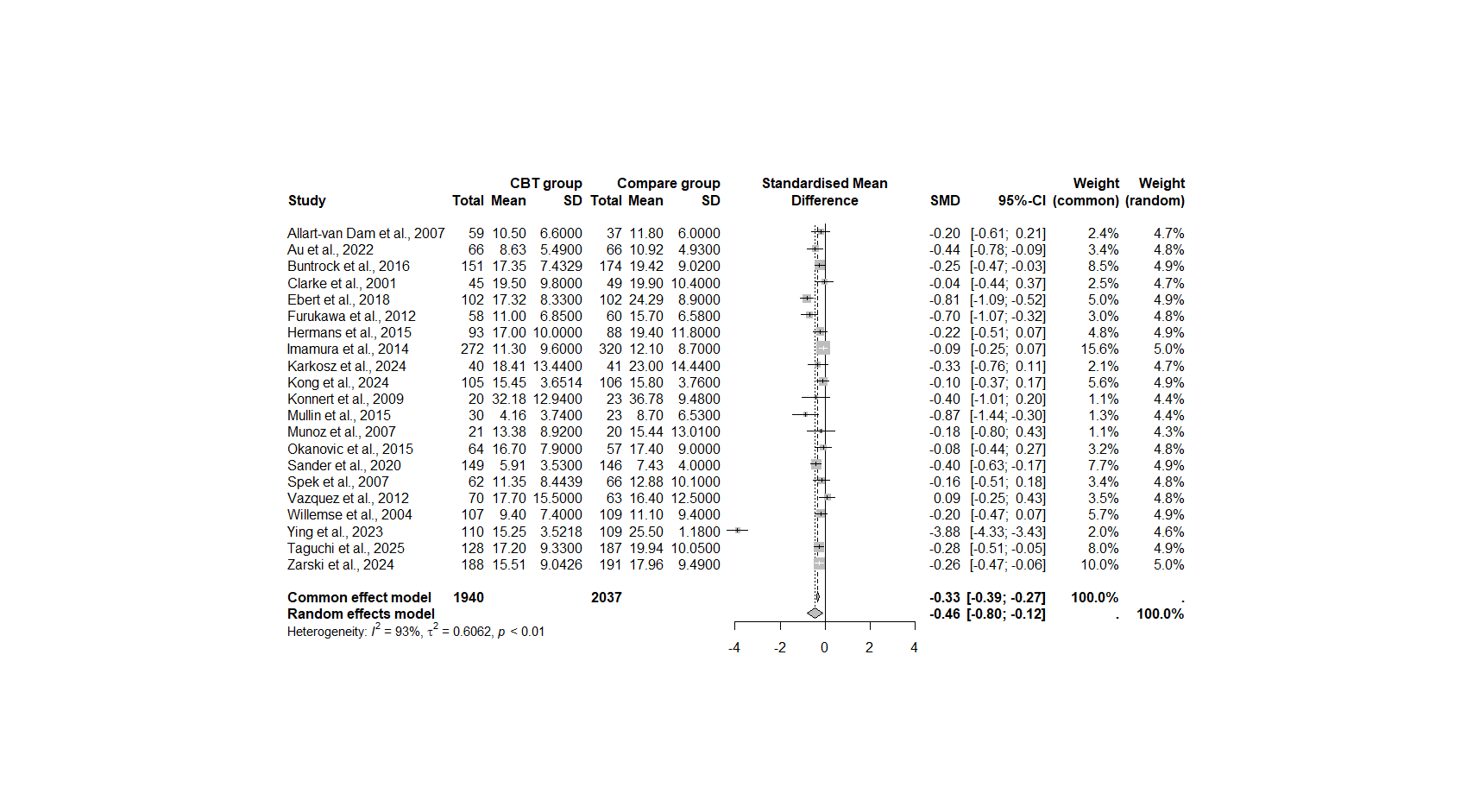** |
| Note. CI = confident interval; SMD = standard mean difference. |

| **Supplementary – Figure 7.** Sensitive Analysis After the removal of both Chen et al. 2025 and Ying et al. 2023 |
| --- |
| **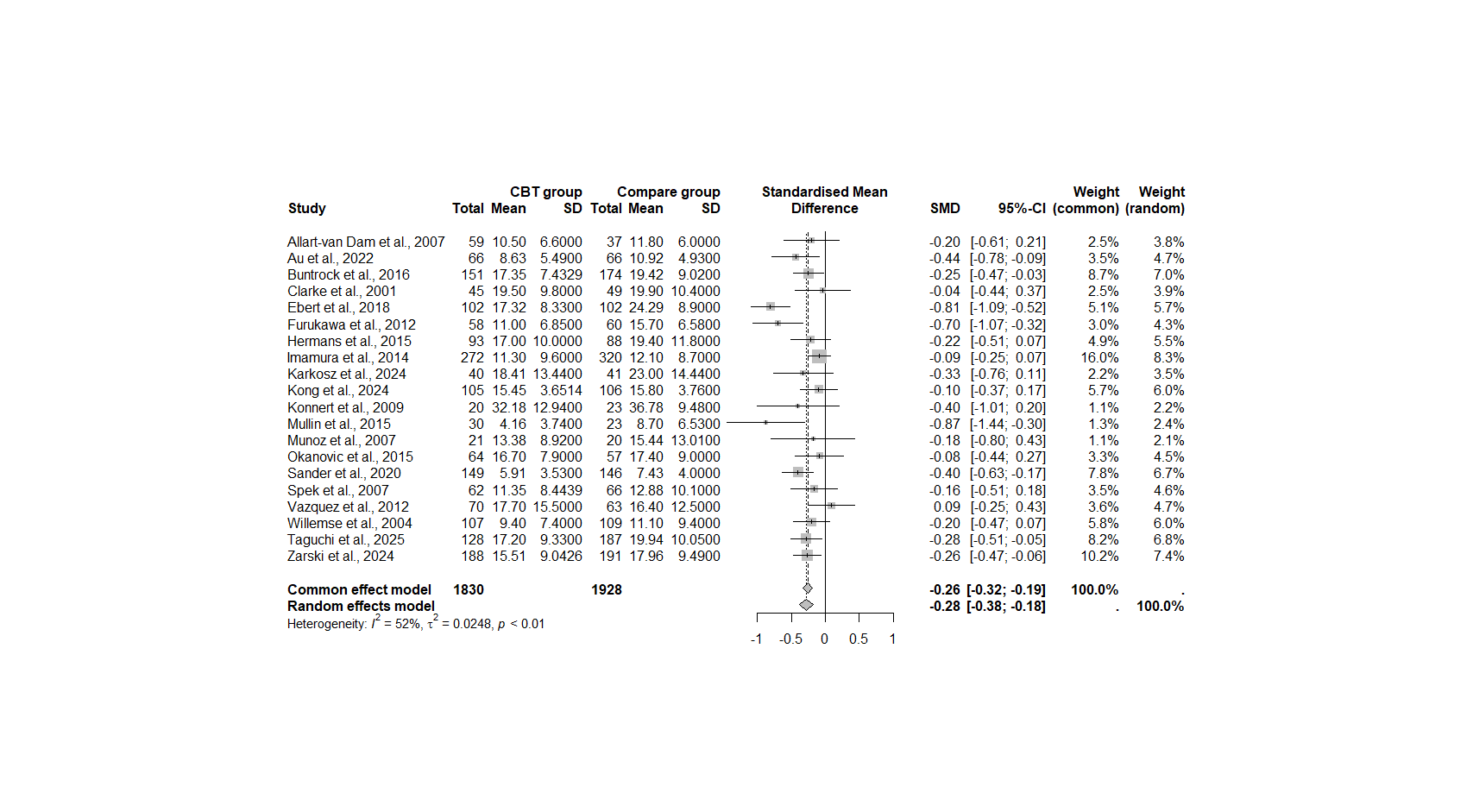** |
| Note. CI = confident interval; SMD = standard mean difference. |
